# Neonatal hyperthyroidism with myocardial injury: a case report

**DOI:** 10.3389/fped.2026.1767859

**Published:** 2026-02-19

**Authors:** Tian Junhua, Jiang Qiuyan, Liu Ranfeng

**Affiliations:** 1 Department of Pediatrics, Ningbo Zhenhai People’s Hospital, Ningbo, Zhejiang, China; 2 Department of Endocrinology, Ningbo Zhenhai People’s Hospital, Ningbo, Zhejiang, China

**Keywords:** case report, myocardial injury, neonatal hyperthyroidism, pediatric, therapy

## Abstract

Neonatal hyperthyroidism (NH) is a rarely reported condition, but inappropriate treatment can have serious consequences. Early diagnosis, treatment, and follow-up are, therefore, essential. We present the case of a male newborn who was admitted to the Pediatrics Department 24 min after birth due to prematurity, presenting with tachypnea and grunting for 20 min. Subsequently, the infant developed increased respiratory rate, tachycardia, crying, and irritability. Bilateral periorbital edema and hyperdynamic heart sounds were also noted. Myocardial injury markers were significantly elevated, indicating myocardial injury. Following treatment with dopamine and dobutamine, the heart rate decreased, but crying, irritability, and periorbital edema persisted. Further history-taking led to a diagnosis of NH with neonatal goiter. The infant was started on oral methimazole and propranolol. Three days after initiation of therapy, the symptoms resolved rapidly, and the patient was discharged.

## Introduction

Neonatal Hyperthyroidism (NH) is a rare condition, with an estimated incidence of approximately 1–2 per 50,000 live births (1). NH can be classified as primary or secondary. Primary hyperthyroidism is caused by genetic mutations (2), while secondary NH (85%–90% of cases) most commonly occurs in infants born to mothers with diffuse goiter or thyroiditis (3). Maternal thyrotropin receptor antibody (TR-Ab) can cross the placenta and stimulate the fetus's thyroid gland to overproduce thyroid hormones, leading to NH (4). Although hyperthyroidism is rarely reported in newborns, affected infants may exhibit symptoms as early as the second trimester of pregnancy. These can include increased physiological activity, such as tachycardia, tachypnea, irritability, hyperphagia without sufficient weight gain, and microcephaly. Other possible manifestations are thrombocytopenia, growth retardation, vomiting, and diarrhea (5). Similar to their mothers, newborns may also present with exophthalmos. If a congenital goiter is present, trachea compression may interfere with respiration at birth. A persistently elevated heart rate may progress to heart failure. Untreated hyperthyroidism can result in premature cranial suture closure, intellectual disability, poor growth, and hyperactivity later in childhood (6). For newborns at risk of hyperthyroidism, obstetrics department should promptly identify maternal risk factors, perform appropriate examinations, and communicate relevant maternal history to pediatrics in a timely manner (7). Pediatric departments must closely monitor high-risk infants and initiate immediate treatment if clinical symptoms suggestive of hyperthyroidism appear.

## Case report

**Figure 1:**
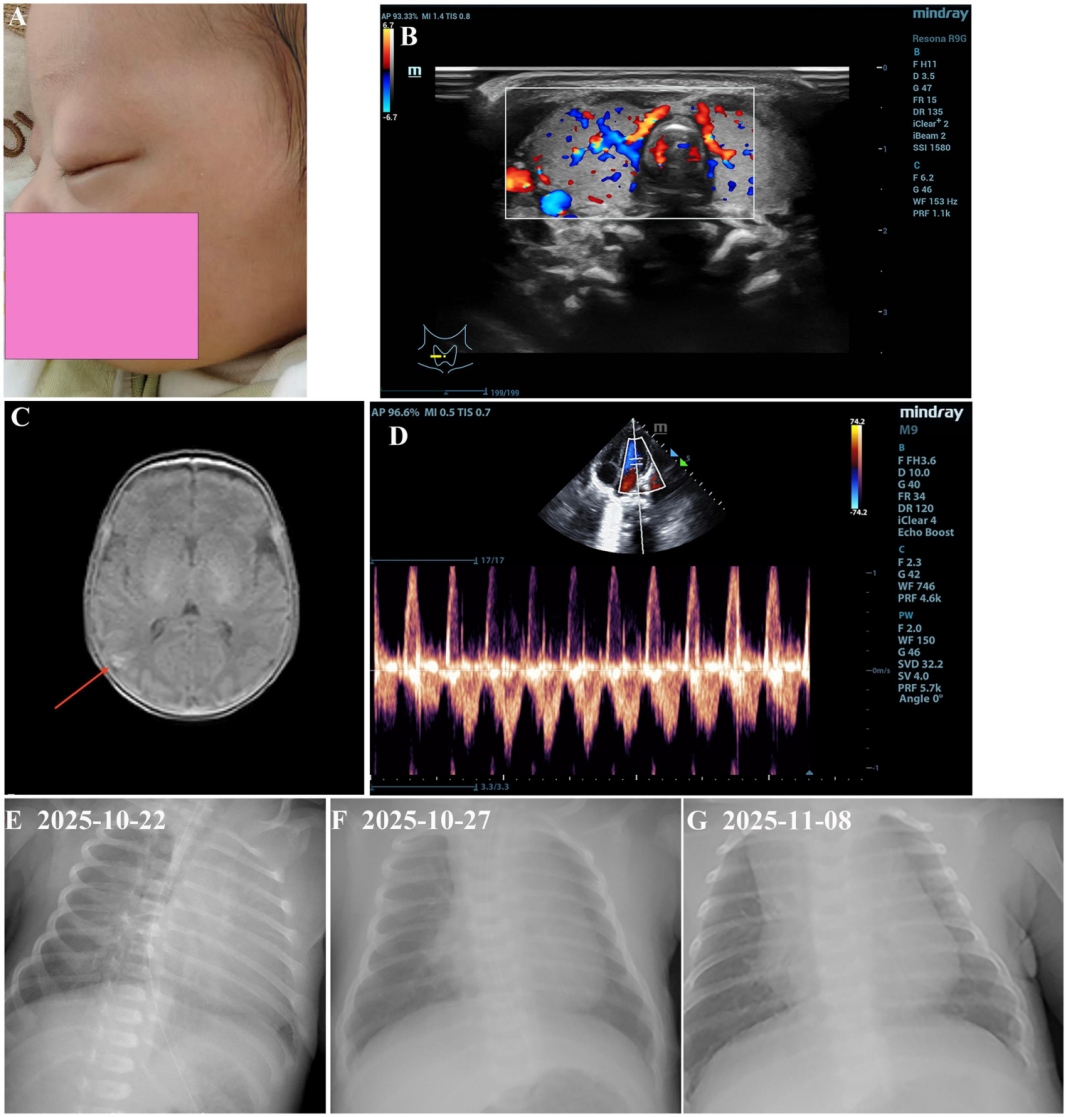
Clinical examination of abnormal indicators with neonatal hyperthyroidism. **(A)** Neonatal hyperthyroidism face has eyelid edema. **(B)** Ultrasound Examination of thyroid gland. Both thyroid glands are enlarged in shape, with smooth, clear and intact capsules. Color Doppler shows abundant blood flow signals in the gland. **(C)** Magnetic resonance imaging of the brain, T1W1 intensive signaling indicate an intra cerebral hemorrhages in the right occipital lobe. **(D)** Ultrasound examination of cardiography. Patent foramen ovale. Chest radiograph of the newborn, obtained on 22 October 2025 **(E)**, 27 October 2025 **(F)**, 8 November 2025 **(G)**, showing thickened lung texture.

A male newborn was admitted to the Pediatric Department 24 min after birth due to prematurity, with tachypnea and grunting for 20 min. The newborn was born vaginally at a gestational age of 34 weeks and 2 days following preterm labor. The infant was the first child of second pregnancy of the mother, with a birth weight of 2.1 kg, length of 46 cm, and head circumference of 30.5 cm (Figure 1A). The amniotic fluid was clear, but a Cord Around the Neck 1 (CAN1) was observed. His Apgar scores were 9 at 1 min and 10 at 5 min.

Physical examination on admission revealed a body temperature of 35.0 °C, a pulse rate of 145 beats/minute, a respiratory rate of 60 breaths/minute, and a blood pressure of 68/46 mmHg. The infant exhibited features of prematurity, a normal mental response, a strong and melodious cry, tachypnea, grunting, and frothing at the mouth. The skin was not fully ruddy and was covered with significant vernix caseosa; no petechiae or rash were observed on the skin or mucous membranes. The anterior fontanelle measured 1 × 1 cm and was normal in tension. A non-fluctuant lump measuring approximately 4 × 3 cm was palpated over the left parietal region. There was no exophthalmos, eyelid retraction, or palpable thyroid enlargement (Figure 1B). Perioral cyanosis was noted without nasal flaring or head-bobbing respiration. The chest showed no deformities, but intercostal, subcostal, and suprasternal retractions were observed during inspiration. Bilateral respiratory movements were symmetrical, with coarse breath sounds audible on both sides and no significant rales. The heart rhythm was regular, and the heart sounds were of medium intensity, with a grade I systolic murmur was heard over the precordium. The umbilical cord was dry without bleeding, discharge, or periumbilical erythema or swelling. The liver and spleen were normal on palpation, and bowel sounds were present. Limb movements were reduced, and primitive reflexes such as rooting and sucking were not elicited. Based on these findings, the infant was diagnosed with respiratory distress, prematurity and low birth weight.

## Treatment course

The neonatal chest radiograph revealed bilateral pulmonary exudative lesions (Figure 1E). The infant received non-invasive ventilation via Nasal Continuous Positive Airway Pressure (NCPAP) from day 1 to day 8, along with penicillin from day 1 to day 6 for infection, intravenous nutritional support and fluid management. Dynamic monitoring of blood gas, electrolytes, blood glucose, and blood pressure revealed that the values remained within normal limits. During the first week after birth, the infant's heart rate (HR) fluctuated between 120 and 160 beats per minute; bedside cardiac ultrasound showed no abnormalities (Figure 1D). Breastfeeding was initiated shortly after delivery. On day 7 of life, NCPAP was discontinued and replaced with nasal catheter oxygen (FiO_2_ 30%, Flow 2 L/min); the respiratory rate remained slightly elevated, and Breastfeeding volume was adequate.

On day 9 of life, the newborn's vital signs changed, with an increased respiratory rate (approximately 65 breaths/min) and mild inspiratory retractions. Transcutaneous oxygen saturation remained normal, while the HR was approximately 160–165 beats per minute. There was no agitation, irritability, screaming, or convulsions. Oxygen delivery was switched from nasal catheter to with non-invasive ventilation via the High-Flow Nasal Cannula (HFNC) mode (FiO_2_ 30%, Flow 4 L/min). Blood pressure, routine blood tests and high-sensitivity C-reactive protein (hsCRP) levels were normal. Blood gas analysis indicated elevated blood lactate. A repeat chest radiograph showed a symmetrical thoracic cage, reduced radiolucency in both lung fields, thickened lung markings, patchy opacities in the right lung, and no cardiac abnormalities (Figure 1F). Ceftazidime (60 mg/kg/d, i.v.d) was administered as antibiotic therapy.

By day 12 of life, although the infant's HR decreased, mild dyspnea persisted. Blood gas analysis revealed hyperkalemia, low partial pressure of oxygen, and elevated lactate. Ventilation was switched back to NCPAP [FiO_2_ 25%, positive end-expiratory pressure, (PEEP6) cmH_2_O], and saline infusion was given. On NCPAP, breathing stabilized, the HR remained between 150 and 164 beats per minute, and no agitation or irritability was noted. Blood pressure and blood glucose levels were normal.

On day 16 of life, the infant again developed tachycardia, with a resting HR of 148–160 beats per minute, rising sharply to 170 beats per minute with minimal activity, accompanied by crying and irritability. Bilateral periorbital edema and hyperdynamic heart sounds were also observed. Myocardial injury markers were significantly elevated (high-sensitivity troponin I, 0.5313 ng/mL, and creatine kinase MB isoenzyme, 11.81 ng/mL), suggesting myocardial injury (defined by an elevated cardiac troponin level above the 99% upper reference limit). To improve microcirculation and reduce cardiac load, continuous micropump infusion of dopamine (3 µg/kg/min) combined with dobutamine (4.5 µg/kg/min) was administered from day 16 to day 21. The HR subsequently decreased to 130–150 beats per minute, but crying, irritability, and periorbital edema persisted (Figure 1A).

On day 18 of life, packed red blood cells were transfused for anemia. Post-transfusion, the symptoms showed no significant improvement (Figure 1G). Further history-taking revealed that the infant's mother had been diagnosed with threatened preterm labor at 34^+1^ weeks of gestation, and had a history of hyperthyroidism for over 5 years. During pregnancy, her blood glucose, fetal heart rate and uterine contractions were monitored, and she was treated with oral methimazole, intravenous hydrochloride, and intermittent oxygen inhalation. Thyroid function tests were urgently performed for the infant, leading to a diagnosis of NH with neonatal goiter. Oral methimazole (1.25 mg) and propranolol (2 mg) were commenced, and dopamine and dobutamine were discontinued. Three days after initiation of this regimen, the symptoms resolved rapidly.

Given the potential side effects of methimazole, including hepatotoxicity and neutropenia, liver function was tested on day 5 of treatment, revealing elevated direct bilirubin, suggesting mild cholestasis. Oxygen therapy was discontinued on day 26 of life. Myocardial injury markers returned to normal, liver function stabilized within the normal range, and thyroid function tests normalized. On day 27 of life, the infant weighed 3310 g and was discharged on medications after receiving health education and arrangements for outpatient follow-up (8).

## Discussion

Neonatal hyperthyroidism (NH) can be life-threatening and typically manifests within the first 10 days of life. Without prompt treatment, the condition may deteriorate rapidly, with a reported mortality rate of 15%–20% (9). In the present case, symptoms of hyperthyroidism appeared on day 9 after birth. The markedly elevated TR-Ab level of 13.27 IU/L (normal <1.75 IU/L) indicated secondary hyperthyroidism. The mother had a confirmed history of hyperthyroidism for over 5 years and was on regular oral methimazole; however, her TR-Ab levels were not monitored during pregnancy. Maternal TR-Ab transferred via the placenta binds to the neonatal TSH receptors, activating the adenylate cyclase-cAMP signaling pathway. This stimulates thyroid hormone synthesis and secretion while suppressing TSH via negative feedback. Higher maternal TR-Ab levels increase the likelihood of NH in the neonate. Therefore, monitoring TR-Ab levels in pregnant women with hyperthyroidism can help predict the occurrence of NH (10). This form of NH usually resolves spontaneously as maternal antibodies are gradually cleared. In contrast, another much rarer type of NH is caused by mutations in the TSH receptor gene and leads to persistent disease (11).

The clinical presentation of NH is highly variable. It frequently occurs in preterm or low birth weight infants. In the current case, the infant developed tachycardia (148–160 beats/min), irritability, crying, eyelid edema, and exophthalmos around day 10 (12). Additionally, cranial MRI on day 27 revealed a small hemorrhagic focus in the right occipital lobe (Figure 1C), consistent with previously reported NH manifestations. However, unlike the thrombocytopenia commonly described in NH, this infant did not develop severe thrombocytopenia.

According to previous literature, NH may present symptoms resembling congenital heart disease, such as tachycardia, cardiac insufficiency, and heart murmurs (13). In the present case, however, the rare feature of myocardial injury emerged as early as day 16 after birth, with laboratory tests showing markedly elevated cardiac markers. The heart is a major target organ for thyroid hormones, and they influence cardiac physiology through several mechanisms (14): (1) In hyperthyroidism, increased myocardial Na^+^-K^+^-ATPase activity alters transmembrane ion fluxes, affecting cardiomyocyte electrophysiology; (2) Thyroid hormone effects on the cardiac conduction system can promote lymphocyte infiltration, focal necrosis, and fibrosis, potentially leading to conduction block; and (3) Excess thyroid hormone enhances sympathetic tone, upregulates myocardial β-adrenergic receptors, and increases sensitivity to catecholamines, resulting in tachycardia and elevated myocardial oxygen consumption.

Notably, elevated cardiac troponin and B-type natriuretic peptide (BNP) levels may be associated with acute respiratory distress (15, 16). The infant was initially admitted with respiratory distress, and a chest radiograph showed bilateral pulmonary exudative lesions. The mechanisms linking acute respiratory distress to elevating cardiac markers include systemic inflammatory damage triggered by the systemic inflammatory response syndrome (SIRS). Severe pulmonary inflammation releases large amounts of pro-inflammatory cytokines (such as TNF-α and IL-6), which can induce a systemic inflammatory response that directly injures cardiomyocytes, increasing myocardial permeability and the release of myocardial markers (17). Furthermore, inflammation, hypoxia, and oxidative stress can collectively promote mitochondrial dysfunction, apoptosis, or necrosis in myocardial cells, leading to the release of troponin T/I and BNP (18).

Mild or asymptomatic NH may not require specific treatment (12). In severe cases, propylthiouracil (PTU) or methimazole is used as the first-line oral therapy. In this case, early hyperthyroid symptoms were subtle, and methimazole was initiated after symptoms worsened. Due to tachycardia, the β-blocker propranolol was co-administered. Symptoms improved markedly within three days. By day 26, all clinical laboratory parameters had normalized, and the infant was discharged on day 27.

In conclusion, NH complicated by myocardial injury is relatively uncommon in clinical practice. Pediatric departments should obtain a thorough maternal history, closely monitor high-risk newborns, and intervene promptly.

## Data Availability

The original contributions presented in the study are included in the article/Supplementary Material, further inquiries can be directed to the corresponding author.
