# Supplementary material for: Neonatal hyperthyroidism with myocardial injury: a case report
Source: Front Pediatr. 2026 Feb 19;14:1767859. doi: 10.3389/fped.2026.1767859 (PMC12960539; doi:10.3389/fped.2026.1767859)
Supplement: Supplementary file 1 [file Supplementaryfile1.pdf]

Prenatal-related information：

1）Pregnant woman's **electrocardiogram**

宁波市镇海区人民医院医疗集团心电中心

总院　　心电图检查报告单

姓名： [redacted]　　出生日期： 1989-05-10　　门/急诊号：2025102101294　　科室：　产科门诊

性别： 女　　床号：　　住院号：　　检查号：NBQY202545728

心　率：	93　次/分	P-R：	117　ms	P：	78　ms	QT：	310　ms	QTc：	386　ms
QRS电轴：	+13°	QRS：	79　ms						

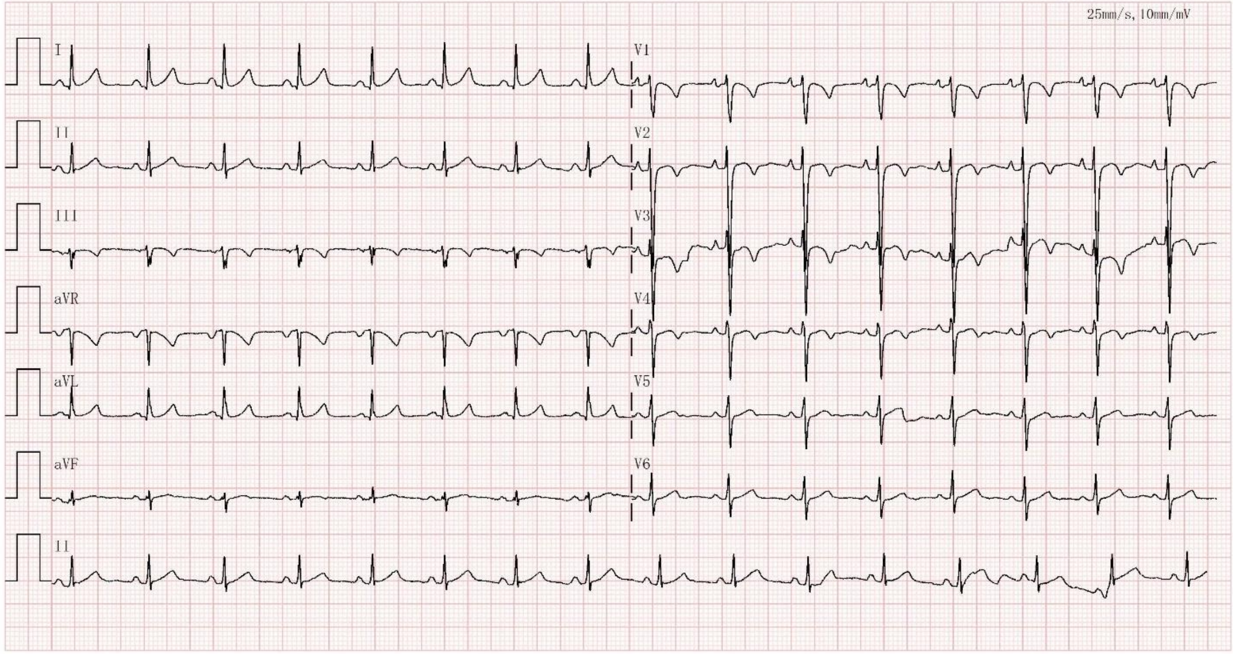

诊断提示：　窦性心律

报告医生：

检查时间： 2025-10-21 14:57:56

本报告仅供临床医生参考，报告医生签名有效

报告时间： 2025-10-21 14:58:14

2）Pregnant woman's **thyroid function tests**

甲状腺功能常规七项=

宁波市镇海区人民医院(宁波市第七医院)检验报告单

姓名: [redacted] 住院号: 25025086 样 本: 血清 样号: 11

性别: 女 病 区: 1-6病区 申请医生: 许丹丹 条码: 152002647427

出生日期: 1989/05/10 床 号: 2108 临床诊断: 先兆早产不伴分娩,孕2产0 标本状态: 标本合格

NO	检验项目	结果		参考区间	单位	实验方法
1	*总三碘甲状腺原氨酸	2.08	↑	0.66－1.61	μg/L	贝克曼化学发光
2	*游离三碘甲状腺原氨酸	7.29	↑	3.28－6.47	pmol/L	贝克曼化学发光
3	*总甲状腺素	160.01	↑	54.40－118.50	μg/L	贝克曼化学发光
4	*游离甲状腺素	16.79	↑	7.64－16.03	pmol/L	贝克曼化学发光
5	*促甲状腺激素	0.00	↓	0.56－5.91	mIU/L	贝克曼化学发光
6	*甲状腺球蛋白抗体	0.0		<4.9	IU/mL	贝克曼化学发光
7	*抗甲状腺过氧化物酶抗体	21.9	↑	<9.0	IU/mL	贝克曼化学发光

Beckman Chemiluminescence

1. 总三碘甲状腺原氨酸 (**Total Triiodothyronine，T3**)
2. 游离三碘甲状腺原氨酸 (**Free Triiodothyronine， FT3**)
3. 总甲状腺素 (**Total Thyroxine， T4**)
4. 游离甲状腺素 (**Free Thyroxine， FT4**)
5. 促甲状腺激素 (**Thyroid Stimulating Hormone， TSH**)
6. 甲状腺球蛋白抗体 (**Thyroglobulin Antibody , Tg**)
7. 抗甲状腺过氧化物酶抗体 (**Anti-Thyroid Peroxidase Antibody , TPOAb**)

采集时间: 25/10/23 06:24 核收时间: 25/10/23 07:52 报告时间: 25/10/23 09:19

本检验结果仅反映此检验标本信息 仪 器: DM2LINK_DXI800 检 验 者: 邢桂生 报 告 者: [illegible]

地址: 宁波市镇海区骆驼街道南二西路718号 电话: 86655021(检验科) *: 浙江省互认项目标识

3）Pregnant woman's **blood routine tests**

血常规+超敏CRP

宁波市镇海区人民医院(宁波市第七医院)检验报告单

急

姓名: [redacted]　住院号: 25025086　样　本: 全血　样号: 65

性别: 女　病　区: 1-6病区　申请医生: 许丹丹　条码: 152002646787

出生日期: 1989/05/10　床　号: 2108　临床诊断: 先兆早产不伴分娩　标本状态: 标本合格

	检验项目	结果		参考区间	单位
High-sensitivity C-Reactive Protein	超敏C反应蛋白	<0.5		<8.0	mg/L
White Blood Cell Count (WBC)	*白细胞计数	11.4	↑	3.5-9.5	×10^9/L
Lymphocyte % (Lymphocytes %)	淋巴细胞%	11.8	↓	20.0-50.0	%
Monocytes % (Monocytes %)	单核细胞%	6.0		3.0-10.0	%
Neutrophils %	中性粒细胞%	82.0	↑	40.0-75.0	%
Eosinophils %	嗜酸性粒细胞%	0.1	↓	0.4-8.0	%
Basophils %	嗜碱性粒细胞%	0.1		0.0-1.0	%
Lymphocyte Count	淋巴细胞绝对数	1.35		1.10-3.20	×10^9/L
Monocyte Count	单核细胞绝对数	0.68	↑	0.10-0.60	×10^9/L
Neutrophils Count	中性粒细胞绝对数	9.37	↑	1.80-6.30	×10^9/L
Eosinophils Count	嗜酸性粒细胞绝对数	0.01	↓	0.02-0.52	×10^9/L
Basophils Count	嗜碱性粒细胞绝对数	0.01		0.00-0.06	×10^9/L
Red Blood Cell Count (RBC)	*红细胞计数	4.78		3.80-5.10	×10^12/L
Hemoglobin (Hb/HGB)	*血红蛋白	122		115-150	g/L
Hematocrit / Packed Cell Volume (HCT/PCV)	*红细胞比容	38.90		35.00-45.00	%
Mean Corpuscular Volume (MCV)	*平均红细胞体积	81.4	↓	82.0-100.0	fL
Mean Corpuscular Hemoglobin (MCH)	*平均血红蛋白含量	25.5	↓	27.0-34.0	pg
Mean Corpuscular Hemoglobin Concentration (MCHC)	*平均血红蛋白浓度	314	↓	316-354	g/L

检验项目	结果	参考区间	单位	
红细胞分布宽度	13.0	11.0-14.5	%	Red Cell Distribution Width (RDW)
*血小板计数	145	125-350	×10^9/L	Platelet Count (PLT)
血小板平均体积	11.1	6.5-13.0	fL	Mean Platelet Volume (MPV)
血小板分布宽度	15.2	9.0-18.1	%	Platelet Distribution Width (PDW)
血小板比容	0.16	0.11-0.28	%	Platelet hematocrit

采集时间: 25/10/21 15:46　核收时间: 25/10/21 16:06　报告时间: 25/10/21 16:17

本检验结果仅反映此检验标本信息　仪 器: 急诊血常规　检 验 者: 杨明鑫　报 告 者: [illegible]

地址: 宁波市镇海区骆驼街道南二西路718号　电话: 86655021(检验科)　方法学: 阻抗法+流式计数　*: 浙江省互认项目标识

4) Pregnant woman's **liver and kidney function tests**

血清总胆汁酸⊖肝功
肾功心肌酶血糖血脂

宁波市镇海区人民医院(宁波市第七医院)检验报告单

姓名: [redacted]　住院号: 25025086　样　本: 血清　样号: **90**

性别: 女　病　区: 1-6病区　申请医生: 许丹丹　条码: 152002646782

出生日期: 1989/05/10　床　号: **2108**　临床诊断: 先兆早产不伴分娩　标本状态: 标本合格

	检验项目	结果		参考区间	单位
Total Protein (TP)	*总蛋白	61.7	↓	65.0－85.0	g/L
Albumin	*白蛋白	36.2	↓	40.0－55.0	g/L
Globulin	球蛋白	25.5		20.0－40.0	g/L
Albumin/Globulin	白球比例	1.42		1.20－2.40	
Alanine Aminotransferase (ALT)	*丙氨酸氨基转移酶	20		7－40	U/L
Aspartate Aminotransferase (AST)	*天门冬氨酸氨基转移酶	17		13－35	U/L
Total Bilirubin	*总胆红素	7.6		0.0－23.0	μmol/L
Direct Bilirubin	*直接胆红素	1.4		0.0－4.0	μmol/L
Indirect Bilirubin	间接胆红素	6.2		0.0－19.0	μmol/L
Alkaline Phosphatase (ALP)	*碱性磷酸酶	261	↑	35－100	U/L
γ-Glutamyl Transpeptidase (GGT)	*γ-谷氨酰基转移酶	8		7－45	U/L
Cholinesterase	*胆碱酯酶	5353		5000－12000	U/L
Total bile acids	*总胆汁酸	2.8		0.0－15.0	μmol/L
Blood Urea Nitrogen (BUN)	*尿素	3.60		2.90－8.20	mmol/L
Creatinine (Cr)	*肌酐	30.0	↓	41.0－73.0	μmol/L
Estimated Glomerular Filtration Rate	肾小球滤过率(eGFR)	141.9		>90	mL/min/
Uric Acid (UA)	*尿酸	336.8		155.0－357.0	μmol/L
Glucose	*葡萄糖	4.67		3.89－6.11	mmol/L
Triglycerides	*甘油三酯	2.25	↑	0.00－1.70	mmol/L
Total Cholesterol	*总胆固醇	4.94		3.00－5.70	mmol/L
High-density lipoprotein cholesterol	*高密度脂蛋白胆固醇	2.06		>1.04	mmol/L

检验项目	结果		参考区间	单位	
*低密度脂蛋白胆固醇	2.23		1.89－4.21	mmol/L	Low-density lipoprotein cholesterol
*载脂蛋白E	47		30－60	mg/L	Apolipoproteins E
脂蛋白a	31.70		0.00－300.0	mg/L	Lipoproteins a
*肌酸激酶	18	↓	26－140	U/L	Creatine Kinase (CK)
*乳酸脱氢酶	164		120－250	U/L	Lactate Dehydrogenase (LD)
*钾	4.36		3.50－5.30	mmol/L	Potassium
*钠	138		137－147	mmol/L	Sodium
*氯	105		99-110	mmol/L	Chloride
*游离脂肪酸	0.92	↑	0.10－0.90	mmol/L	Free fatty acids
*血清前白蛋白	235		200－400	mg/L	Serum prealbumin

采集时间: 25/10/21 15:46　核收时间: 25/10/22 08:20　报告时间: 25/10/22 09:31

本检验结果仅反映此检验标本信息　仪　器: DM2LINK_AU5800　检　验　者: 邢桂生　报　告　者: 孔亢娜

地址: 宁波市镇海区骆驼街道南二西路718号　电话: 86655021(检验科)　*: 浙江省互认项目标识

5) The infant’s temperature Chart

Temprature Chart

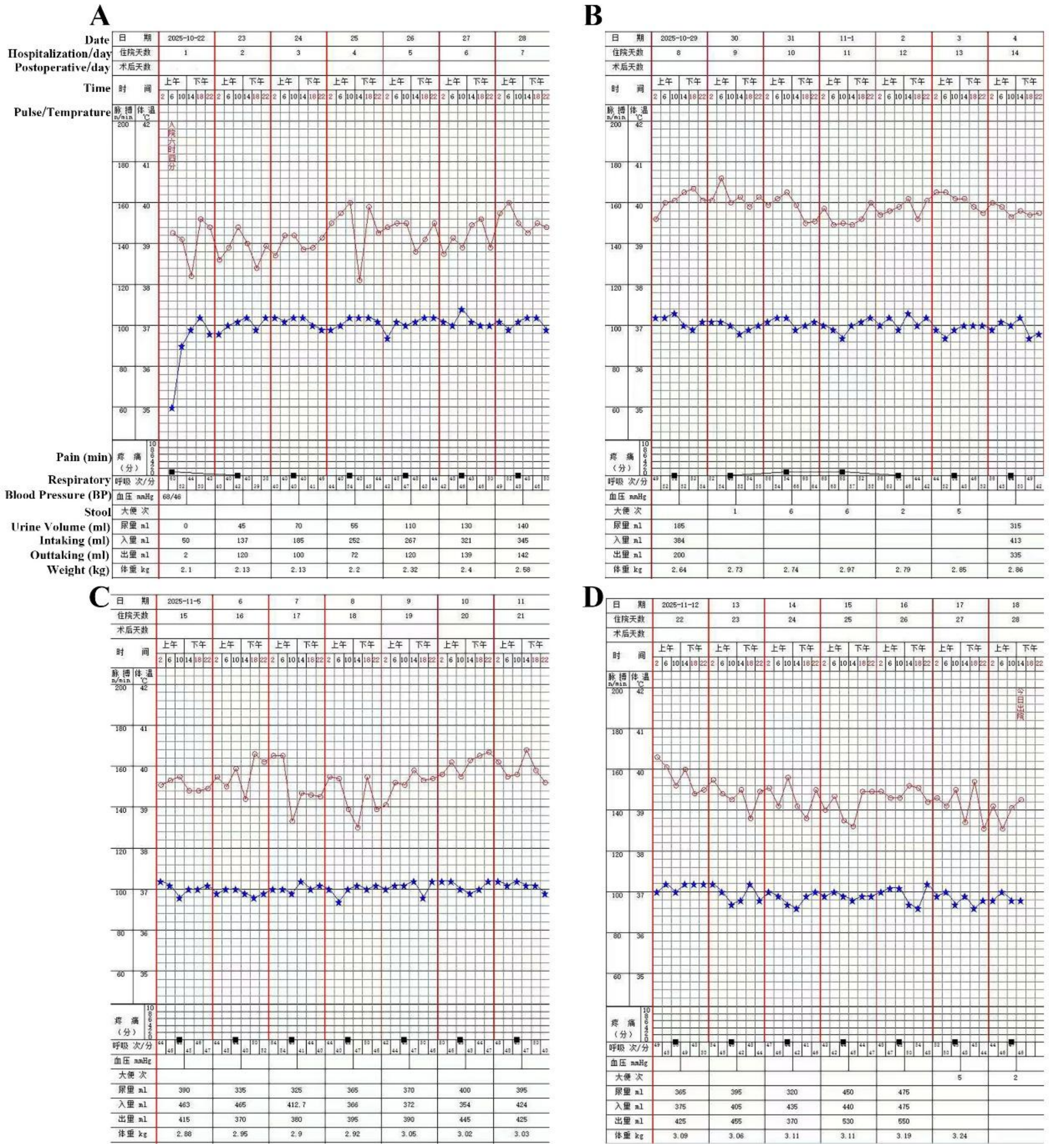

6) The infant's myocardial enzymes, troponin

急诊心肌损伤标志物=

宁波市镇海区人民医院(宁波市第七医院)检验报告单

急

姓名:[redacted] 住院号: 25025103 样 本:血浆 2025.11.6 样号:42

性别:男 病 区:新生儿病区 申请医生:田俊华 条码:152002663924

出生日期: 2025/10/22 床 号:2008 临床诊断:新生儿高胆红素血症,新生 标本状态:标本合格

NO	检验项目	结果		参考区间	单位	实验方法
1	*高敏肌钙蛋白I	0.5313	↑↑	<0.0175	ng/mL	贝克曼化学发光
2	*肌红蛋白	62.7		0.0-70.0	ng/mL	贝克曼化学发光
3	*肌酸激酶同工酶-MB	11.81	↑	0.30-4.00	ng/mL	贝克曼化学发光

Beckman Chemiluminescence

1. High-sensitivity troponin I
2. Myoglobin
3. Creatine Kinase, MB Isoenzyme

2025.11.10

NO	检验项目	结果		参考区间	单位	实验方法
1	*高敏肌钙蛋白I	0.4941	↑	<0.0175	ng/mL	贝克曼化学发光
2	*肌红蛋白	52.8		0.0-70.0	ng/mL	贝克曼化学发光
3	*肌酸激酶同工酶-MB	13.38	↑	0.30-4.00	ng/mL	贝克曼化学发光

2025.11.17

NO	检验项目	结果		参考区间	单位	实验方法
1	*高敏肌钙蛋白I	0.4176	↑	<0.0175	ng/mL	贝克曼化学发光
2	*肌红蛋白	10.9		0.0-70.0	ng/mL	贝克曼化学发光
3	*肌酸激酶同工酶-MB	5.91	↑	0.30-4.00	ng/mL	贝克曼化学发光

2025.12.23

NO	检验项目	结果		参考区间	单位	实验方法
1	*高敏肌钙蛋白I	0.0697	↑	<0.0175	ng/mL	贝克曼化学发光
2	*肌红蛋白	21.9		0.0-70.0	ng/mL	贝克曼化学发光
3	*肌酸激酶同工酶-MB	11.50	↑	0.30-4.00	ng/mL	贝克曼化学发光

急诊B型纳尿肽(BNP)

宁波市镇海区人民医院(宁波市第七医院)检验报告单

急诊

姓名:[redacted] 住院号: 25025103 样 本:血浆 2025.11.6 样号:41

性别:男 病 区:新生儿病区 申请医生:田俊华 条码:152002663925

出生日期: 2025/10/22 床 号:2008 临床诊断:新生儿高胆红素血症,新生儿标本状态:标本合格

NO	检验项目	结果		参考区间	单位	实验方法
1	B型纳尿肽(BNP)	454.0	↑	0.0—100.0	pg/mL	贝克曼化学发光

B-type natriuretic peptide

2025.11.11

NO	检验项目	结果		参考区间	单位	实验方法
1	B型纳尿肽(BNP)	330.0	↑	0.0—100.0	pg/mL	贝克曼化学发光

7) The infant’s thyroid function tests

甲状腺功能常规(五项)

宁波市镇海区人民医院(宁波市第七医院)检验报告单

姓名:[redacted]　住院号: 25025103　样　本:血清　样号:153

性别:男　病　区:新生儿病区　申请医生:刘鑫　条码:152002676867

出生日期: 2025/10/22　床　号:2008　临床诊断:新生儿高胆红素血症,新生　标本状态:标本合格

2025.11.17

NO	检验项目	结果		参考区间	单位	实验方法
1	*总三碘甲状腺原氨酸	1.26		0.66－1.61	μg/L	贝克曼化学发光
2	*游离三碘甲状腺原氨酸	5.30		3.28－6.47	pmol/L	贝克曼化学发光
3	*总甲状腺素	78.72		54.40－118.50	μg/L	贝克曼化学发光
4	*游离甲状腺素	12.89		7.64－16.03	pmol/L	贝克曼化学发光
5	*促甲状腺激素	0.00	↓	0.56－5.91	mIU/L	贝克曼化学发光

2025.11.25

NO	检验项目	结果		参考区间	单位	实验方法
1	*总三碘甲状腺原氨酸	1.22		0.66－1.61	μg/L	贝克曼化学发光
2	*游离三碘甲状腺原氨酸	4.94		3.28－6.47	pmol/L	贝克曼化学发光
3	*总甲状腺素	51.90	↓	54.40－118.50	μg/L	贝克曼化学发光
4	*游离甲状腺素	7.18	↓	7.64－16.03	pmol/L	贝克曼化学发光
5	*促甲状腺激素	0.05	↓	0.56－5.91	mIU/L	贝克曼化学发光

2025.12.09

NO	检验项目	结果		参考区间	单位	实验方法
1	*总三碘甲状腺原氨酸	1.31		0.66－1.61	μg/L	贝克曼化学发光
2	*游离三碘甲状腺原氨酸	5.57		3.28－6.47	pmol/L	贝克曼化学发光
3	*总甲状腺素	51.07	↓	54.40－118.50	μg/L	贝克曼化学发光
4	*游离甲状腺素	9.12		7.64－16.03	pmol/L	贝克曼化学发光
5	*促甲状腺激素	1.15		0.56－5.91	mIU/L	贝克曼化学发光

2025.12.23

NO	检验项目	结果		参考区间	单位	实验方法
1	*总三碘甲状腺原氨酸	1.57		0.66－1.61	μg/L	贝克曼化学发光
2	*游离三碘甲状腺原氨酸	6.12		3.28－6.47	pmol/L	贝克曼化学发光
3	*总甲状腺素	55.79		54.40－118.50	μg/L	贝克曼化学发光
4	*游离甲状腺素	8.81		7.64－16.03	pmol/L	贝克曼化学发光
5	*促甲状腺激素	4.45		0.56－5.91	mIU/L	贝克曼化学发光

2026.01.09

NO	检验项目	结果		参考区间	单位	实验方法
1	*总三碘甲状腺原氨酸	1.45		0.66－1.61	μg/L	贝克曼化学发光
2	*游离三碘甲状腺原氨酸	6.68	↑	3.28－6.47	pmol/L	贝克曼化学发光
3	*总甲状腺素	69.29		54.40－118.50	μg/L	贝克曼化学发光
4	*游离甲状腺素	12.76		7.64－16.03	pmol/L	贝克曼化学发光
5	*促甲状腺激素	3.01		0.56－5.91	mIU/L	贝克曼化学发光

1. 总三碘甲状腺原氨酸 (**Total Triiodothyronine，T3**)
2. 游离三碘甲状腺原氨酸 (**Free Triiodothyronine， FT3**)
3. 总甲状腺素 (**Total Thyroxine， T4**)
4. 游离甲状腺素 (**Free Thyroxine， FT4**)
5. 促甲状腺激素 (**Thyroid Stimulating Hormone， TSH**)
